# 

**Published:** 1890-04-12

**Authors:** 


SATURDAY, APRIL 12th, 1890.

**Science and a Future Life** - 17

**Words of Consolation.**—Truth - 18

**The Doctor's Pulpit.**—Cares and Coffins - 19

**The Dangers of Kissing** - 20

**Everybody's Page** - 21

**Notes and News** - 22

**Annotations.**—Sausages and Polonies—Mr. Lilly and Mr. Herbert Spencer—Was it Hypnotism? - 24

**A Visit to the Hospital at Zurich University** - 27

**April** - 28

**The Practitioner's Mirror and Retrospect:**

MEDICINE—Typhlitis—Acute Tonsilitis—Histeria - 25

SURGERY—The Connection of Burns with Ulceration of the Duodenum—Enlarged Glands - 26

NEW DRUGS, APPLIANCES, AND THINGS MEDICAL—"The Automatic Respirator" - 26

THE PRACTITIONER'S BOOKSHELF—The Pulse - 27

**Passing Topics.**—The Sanitary Condition of the London Hospital - 30

**Scraps and Gleanings** - 30

### EXTRA SUPPLEMENT—THE NURSING MIRROR.

**En Passant.**—A Dramatic Entertainment—Haggerston District Home—Bradford News—Bristol and Clifton Institution—Thousands of Pounds for the National Pension Fund—Lady Roberts' Homes—Bradford Nurses' Institution - v

**The Late Mr. Junius Morgan** - vi

**Everybody's Opinion.**—The Southsea Home—Male Nurses—Uniforms for June - vii

**Love** - vii

**Appointments** - vii

**Lectures** - viii

**Presentation** - viii

**Notes and Queries** - viii

**Vacancies** - viii

**Amusements and Relaxation** - viii

